# Large-bore Aspiration Thrombectomy with the FlowTriever System for the Treatment of Pulmonary Embolism: A Large Single-Center Retrospective Analysis

**DOI:** 10.1007/s00270-024-03819-5

**Published:** 2024-08-07

**Authors:** Travis Pebror, Adam William Schmitz, Andrew Gauger, Reid Masterson, Sabah David Butty

**Affiliations:** 1 Department of Radiology and Imaging Sciences, Indiana University School of Medicine, Indianapolis, IN USA; 2 Indiana University Health Radiology, 714 N Senate Ave, Indianapolis, IN 46202 USA

**Keywords:** Pulmonary embolism, Large-bore thrombectomy, FlowTriever, Catheter-directed intervention

## Abstract

**Purpose:**

Evaluate the outcomes of patients undergoing large-bore aspiration thrombectomy for the treatment of pulmonary embolism at a large university medical center.

**Materials and methods:**

All patients treated for pulmonary embolism with the FlowTriever System (Inari Medical, Irvine, CA) between September 2019 and January 2023 were retrospectively analyzed. The primary safety and effectiveness outcomes included 7- and 30-day all-cause mortality, major bleeding, procedure-associated clinical decompensation, pulmonary vascular or cardiac injury, and pulmonary artery pressure reduction. Additional outcomes included technical success (completing thrombectomy with the device as intended), changes in hemodynamics and supplemental oxygen requirements, and postprocedural intensive care unit stay.

**Results:**

A total of 286 patients were identified. The mean age was 60.5 years, and 90.9% of patients presented with intermediate-risk pulmonary embolism. Technical success was achieved in 96.9% (*n* = 277) of cases. The average reduction in mean pulmonary arterial pressure was 6.8 mmHg, from 28.7 ± 9.0 to 21.9 ± 8.0 mmHg (*p* < 0.0001). Two major bleeds (0.7%), 2 pulmonary vascular injuries (0.7%), and 4 (1.4%) procedure-associated decompensations were reported, but no device-related deaths occurred. The mean post-procedure intensive care unit stay was 2.0 ± 4.1 days, and 49.3% of patients had no postprocedural intensive care unit admittance. The overall 7-day and 30-day all-cause mortality rates were 2.4% and 6.7%, respectively, with a 30-day pulmonary embolism-related mortality rate of 3.5%.

**Conclusion:**

This non-industry-sponsored single-center analysis of large-bore aspiration thrombectomy in a large population corroborates the findings of other studies and confirms that this approach is safe and effective for the treatment high- and intermediate-risk pulmonary embolism.

**Level of Evidence IV:**

Retrospective observational study.

**Graphical Abstract:**

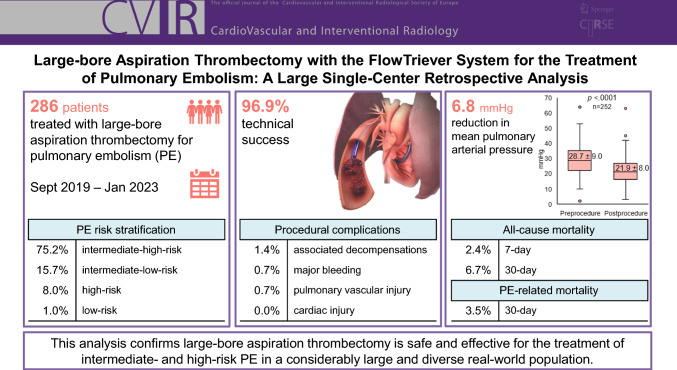

## Introduction

Pulmonary embolism (PE) is associated with significant mortality, representing the third leading cause of cardiovascular death. In-hospital mortality for high-risk PE is often reported above 25% [1–4], while 30-day mortality in intermediate-risk PE is reported near 10% [1, 2]. Current treatment guidelines recommend reperfusion treatment with systemic thrombolytic therapy for high-risk PE and front-line anticoagulation therapy for intermediate-risk PE, escalating to reperfusion treatment in the event of hemodynamic deterioration [5]. However, updated consensus suggests a Pulmonary Embolism Response Team (PERT) also convenes as soon as possible to consider reperfusion treatment, including via catheter-directed therapies, for intermediate-risk PE patients who fail to improve after 24–48 h of therapeutic-dose anticoagulation [6].

The FlowTriever System (Inari Medical, Irvine, CA) is a large-bore aspiration thrombectomy (LBAT) device used for percutaneous PE treatment without thrombolytics. It is the most investigated mechanical thrombectomy treatment for PE, having been evaluated in the industry-sponsored FLARE, FLASH, and FLAME studies with outcomes reported from nearly 1,000 total patients with intermediate- and high-risk PE [7–10]. However, many non-industry-sponsored evaluations of this LBAT device are of modest size [11–14]. Considering the large number of PE patients treated with LBAT at our center, we aimed to evaluate the safety and effectiveness of LBAT in routine clinical practice at our institution through a retrospective review of patients treated with this approach.

## Materials and Methods

### Study Design and Population

Electronic health records of patients treated for PE at a large university medical center between September 2019 and January 2023 were retrospectively analyzed. By September 2019, LBAT had largely supplanted catheter-directed thrombolysis (CDT) as the primary interventional PE treatment method at our institution due to immediacy of clinical improvement following a single-session procedure without the need to administer thrombolytics. To assess outcomes with this updated approach, all patients treated with LBAT using the FlowTriever System were included in this analysis, and those treated with CDT were excluded. No other criteria were applied. Patients were treated using an institutional PE treatment protocol which included: 1) imaging via CT, 2) treatment determination by a multidisciplinary Pulmonary Embolism Response Team (PERT) composed of an emergency medicine physician, interventional radiologist, and pulmonary critical care physician, 3) interventional reperfusion with LBAT in appropriate patients per PERT decision, and 4) admission to the progressive care unit following LBAT unless intensive care unit (ICU) admission was needed.

PE risk stratification was performed using the current European Society of Cardiology (ESC) guidelines [5]. Generally, the PERT recommended intermediate-risk PE patients with central bulky thrombus to be treated via an interventional approach, and patients with low-risk or peripheral PE to receive anticoagulation. Risk stratification markers like troponin, brain natriuretic peptide (BNP), and right heart strain, determined independently by diagnostic radiologists using commonly defined imaging findings such as right ventricular-to-left ventricular (RV/LV) ratio > 0.9, were also considered by the PERT in making treatment determinations.

### Procedure Description

At our institution, a typical LBAT procedure course is as follows. The patient is on anticoagulation before arrival to the procedure room. Additional heparin is given to achieve activated clotting time of 250–300 s, and the patient is placed under conscious sedation. LBAT is initiated by gaining vascular access through the femoral vein. A balloon-tipped or pigtail catheter is navigated through the heart into the pulmonary arteries, and pressure measurements may be taken. Once the pulmonary vasculature has been accessed, the catheter is removed over a stiff guidewire to allow for insertion of the Triever aspiration catheter (16 Fr, 20 Fr or 24 Fr). This is positioned immediately proximal to the embolic debris and aspiration is initiated with a 60 mL syringe attached to the Triever catheter side port. Aspiration is repeated as needed. Mechanical disruption of embolic debris with the FlowTriever disks is not used at our institution. Once it became available (August 2021), the FlowSaver blood return system (Inari Medical) was implemented in the majority of LBAT procedures to filter aspirated blood in the syringe and return it to the patient through the access site. The decision of when to terminate the LBAT procedure is left to the discretion of the interventional radiologist performing the procedure based on thrombus removal and observed improvement in clinical status. At the conclusion of the procedure, hemostasis is achieved using a pre-close technique with two Perclose ProGlide devices (Abbott, Plymouth, MN) or a purse-string suture technique.

### Outcomes

The primary safety and effectiveness outcomes assessed included 7- and 30-day all-cause mortality, major bleeding, procedure-associated clinical decompensation, pulmonary vascular or cardiac injury, and pulmonary artery pressure (PAP) reductions. Major bleeding was determined using the Society of Interventional Radiology (SIR) reporting standards, i.e., intracranial, intraocular, or retroperitoneal hemorrhage or any hemorrhage requiring transfusion and/or resulting in a hematocrit decrease ≥ 15% or hemoglobin decrease ≥ 5 g/dL [15]. All clinical decompensation events, defined as an acute physiological worsening of clinical status that posed an immediate increased risk of serious harm or death [16], occurring during hospitalization whether before, during, or after the thrombectomy procedure were recorded. PAPs were measured immediately prior to and following thrombectomy via standard invasive hemodynamic assessment techniques. Additional outcomes included technical success per the SIR reporting standards, i.e., the ability to deliver the device into the pulmonary artery, operate the device to aspirate thrombus, and remove the device at the end of the procedure [15], change in hemodynamic measurements, and postprocedural hospital length of stay (LOS).

### Statistical Analysis

Data values are reported as counts (%) for categorical variables and mean ± standard deviation (SD) or median [interquartile range (IQR)] for continuous variables. For comparative analysis of changes in hemodynamics and oxygenation status following thrombectomy, *p*-values were calculated using two-sided paired Student t tests for continuous data and McNemar tests for categorical data. Change in PAP following thrombectomy was analyzed in patients with paired pre-procedure and post-procedure measurements, and *p*-values were calculated using one-sided paired Student t tests. A *p*-value < 0.05 was considered statistically significant. Statistical analysis was performed in R, version 4.3.2.

### Ethics Statement

This research activity was granted exemption from full Institutional Review Board review and waived informed consent by a qualified staff member of the Human Research Protection Program of the affiliated university in accordance with 45 CFR 164.512(i)(2)(ii).

## Results

### Patient Characteristics and PE Presentation

During the study period, 319 PE patients were treated with catheter-directed therapy, including 33 who underwent CDT and 286 who underwent LBAT. All 286 patients treated with LBAT were included in the analysis population, while the 33 CDT patients were excluded. As depicted in Table 1, the analysis population was 53.5% male, with a mean age of 60.5 ± 16.2 years and a mean BMI of 35.2 ± 10.6 kg/m^2^. Concurrent deep vein thrombosis was present in 62.9% of patients, 18.9% had a history of malignancy, and 16.1% were diagnosed with PE within 3 months of having surgery. Intermediate-high-risk PE was diagnosed in 75.2% of patients, while 15.7% presented with intermediate-low-risk PE, 8.0% with high-risk PE, and 1.0% with low-risk PE. Right ventricle dilatation on CT was identified in 87.4% of the cohort, while 95.5% had elevated cardiac biomarkers.

**Table 1:** Baseline characteristics and pulmonary embolism presentation

Characteristics	*N* = 286
Age, years	60.5 ± 16.2
Male sex	153 (53.5)
BMI, kg/m^2^	35.2 ± 10.6
History of PE or DVT	52 (18.2)
History of PE	31 (10.8)
History of DVT	31 (10.8)
History of PE and DVT	10 (3.5)

Comorbidities and Risk Factors	
Obesity (BMI ≥ 30)	186 (65.0)
Concurrent DVT	180 (62.9)
Hypertension	166 (58.0)
Immobility for ≥ 30 days	74 (25.9)
Current tobacco use	71 (24.8)
Diabetes mellitus	70 (24.5)
History of malignancy	54 (18.9)
Surgery within prior 3 months	46 (16.1)
Current oral contraceptive or estrogen use	14 (4.9)
Pregnancy	3 (1.0)

PE Presentation	
Symptom duration, days	3.4 ± 3.9
High-risk (massive) PE	23 (8.0)
Intermediate-risk (submassive) PE	260 (90.9)
Intermediate-high-risk	215 (75.2)
Intermediate-low-risk	45 (15.7)
Low-risk PE	3 (1.0)
Right ventricle dilatation (RV/LV ratio > 0.9)	250 (87.4)
Elevated troponin or BNP	273 (95.5)
Elevated troponin	264 (92.3)
Elevated BNP	196 (68.5)
Required supplemental oxygen	180 (62.9)
Nasal cannula	136 (47.6)
Mask (any)	33 (11.5)
Ventilator/endotracheal tube	11 (3.8)
Chest pain	152 (53.1)
Hemoptysis	6 (2.1)

Values are mean ± standard deviation or *n* (%)

*BMI* body mass index; *BNP* brain natriuretic peptide; *DVT* deep vein thrombosis; *PE* pulmonary embolism; *RV*/*LV* right ventricle-to-left ventricle diameter ratio

### Procedural Characteristics, Outcomes, and LOS

Table 2 shows the procedural characteristics and LOS data for the cohort. The mean total procedure time from pre-procedure time out to access site closure was 90.5 ± 32.3 min. No patients were treated with thrombolytic therapy intraprocedurally, but 4.5% (*n* = 13) received thrombolytic therapy at an outside hospital prior to the procedure. The mean post-procedural hospital LOS was 7.3 ± 9.1 days and the mean post-procedural ICU LOS was 2.0 ± 4.1 days, with 49.3% of patients not requiring ICU admittance. Among 145 (50.7%) patients with post-procedure ICU admittance, the mean LOS was 3.9 ± 5.1 days.

**Table 2:** Procedural characteristics and length of stay

Characteristic	*N* = 286		
	*n* (%)	Mean ± SD	Median [IQR]
Procedural time, min	–	90.5 ± 32.3	83.0 [71.0, 105.0]
Fluoroscopy time, min^a^	–	23.6 ± 10.6	21.6 [16.9, 28.2]
Technical success^b^	277 (96.9)	–	–
Received thrombolytics during procedure	0.0 (0.0)	–	–
Post-procedure length of stay, days	–	7.3 ± 9.1	5.0 [3.0, 9.0]
Stay on medical floor	–	5.3 ± 6.7	4.0 [2.0, 7.0]
Stay in ICU	–	2.0 ± 4.1	1.0 [0.0, 2.0]
Admitted to ICU post-procedure	145 (50.7)	–	–
Stay in ICU, days^c^	–	3.9 ± 5.1	2.0 [2.0, 4.0]

*ICU* intensive care unit; *IQR* interquartile range; *SD* standard deviation

^a^*n* = 285

^b^Technical success defined as the ability to deliver the device into the pulmonary artery, operate the device to aspirate thrombus, and remove the device at the end of the procedure

^c^Mean ± SD and median [IQR] reported for 145 patients admitted to ICU post-procedure

Technical success was achieved in 277 (96.9%) cases. Of the 9 technical failures, 7 involved emboli that could not be aspirated, 1 procedure could not be completed for undocumented reasons, and 1 procedure was terminated due to a pulmonary artery pseudoaneurysm, details of which are described below. In the 7 cases involving emboli that could not be aspirated, 6 were due to intractable chronic thrombus and 1 was due to very large central thrombus size. Of the 6 patients with intractable chronic thrombus, 1 expired during the procedure and 5 continued medical management with anticoagulation and survived through 30 days. The patient with central thrombus that was too large to aspirate died of uterine hemorrhage in the setting of endometrial cancer 18 days following the procedure.

### Hemodynamics and oxygen supplementation

Table 3 shows changes in hemodynamics and oxygenation status at presentation and post-procedure. Prior to thrombectomy, 183 (64.0%) of patients were tachycardiac (heart rate ≥ 100 beats per minute [bpm]). Of these tachycardiac patients, 54.1% experienced resolution of their tachycardia immediately following the procedure. The mean reduction in heart rate among the entire cohort was 13.5 bpm (*p* < 0.0001). There was not a statistically significant change in the number of patients who required supplemental oxygen prior to and immediately following thrombectomy (*n* = 180 [62.9%] vs *n* = 165 [57.7%]; *p* = 0.0997), though 27.2% of patients with supplemental oxygen requirements at presentation no longer required oxygen support following the procedure.

**Table 3:** Changes in patient hemodynamics and oxygenation status following thrombectomy

Assessments	Presentation* N* = 286	Post-procedure* N* = 286	Mean change	*p*-value^a^
Systolic blood pressure, mmHg^b^	130.0 ± 24.0	123.2 ± 20.1	–7.0	< 0.0001
Diastolic blood pressure, mmHg^b^	84.8 ± 16.9	78.0 ± 15.5	–6.9	< 0.0001
Heart rate, bpm	106.2 ± 20.2	92.9 ± 17.1	–13.5	< 0.0001
Tachycardia (≥ 100 bpm)	183 (64.0)	94 (32.9)	n/a	< 0.0001
Heart rate, bpm^c^	117.6 ± 14.6	98.4 ± 16.1	–19.1	< 0.0001
Resolution of tachycardia^c^	n/a	99 (54.1)	n/a	n/a
Any oxygen supplementation	180 (62.9)	165 (57.7)	n/a	0.0997
Resolution of supplemental oxygen requirement^d^	n/a	49 (27.2)	n/a	n/a

Values are mean ± standard deviation or n (%)

*Bpm* beats per minute

^a^*p*-values calculated using McNemar tests for categorical data and two-sided paired t tests for continuous data

^b^Post-procedure value and mean change calculated for n = 285

^c^Percentage reported out of 183 patients with tachycardia at presentation

^d^Percentage reported out of 180 patients requiring oxygen supplementation at presentation

**Figure 1:**
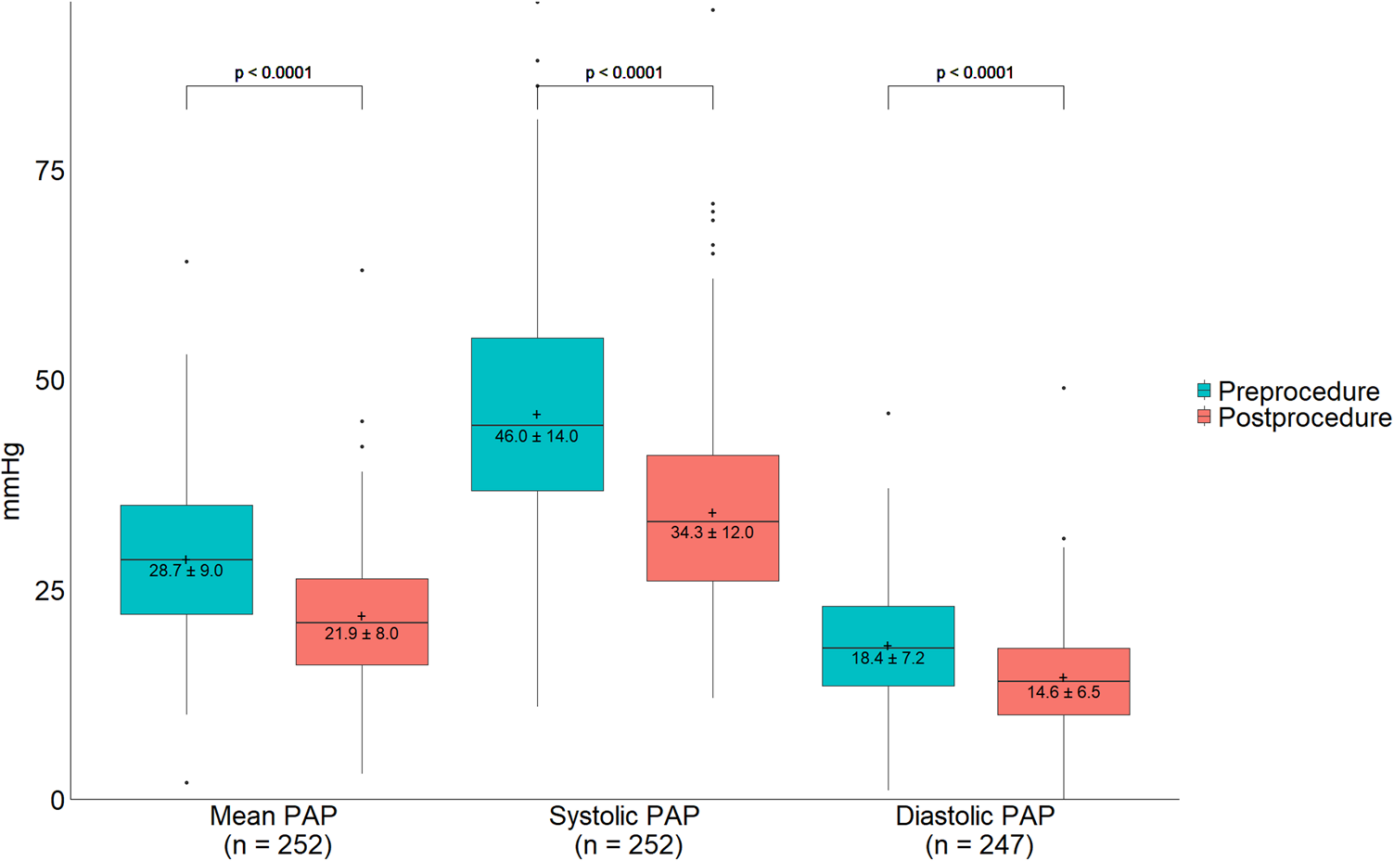
Change in mean pulmonary arterial pressure (PAP), systolic PAP, and diastolic PAP following thrombectomy. Plots and statistics are based on patients with paired pre-procedure and post-procedure measurements (*n* = 247–252). Boxes represent IQR (Q1, Q3) with horizontal bars representing the median and the whiskers representing 1.5 × IQR. Outliers are shown as individual points. The mean is indicated on each box by a “ + ” sign, with numeric mean ± SD data reported below. *P*-values were calculated using one-sided paired *t* test

Figure 1 depicts the change in PAPs among patients with paired pre- and post-thrombectomy measurements. The mean reduction in mean and systolic PAP immediately following thrombectomy was 6.8 mmHg (28.7 ± 9.0 to 21.9 ± 8.0 mmHg; *p* < 0.0001) and 11.7 mmHg (46.0 ± 14.0 to 34.3 ± 12.0; *p* < 0.0001), respectively.

### Safety and Mortality Outcomes

Safety and mortality outcomes for the cohort are shown in Table 4. There were 2 (0.7%) major bleeds consisting of 1 case of abdominal bleeding detected post-procedure likely due to rebleed of a retroperitoneal hematoma and 1 case resulting from intraprocedural laceration of the corona mortis which occurred during sheath insertion and was treated with coil embolization. Two (0.7%) pulmonary vascular injuries occurred: 1 case of contrast extravasation into the mediastinum observed on postprocedural angiogram and 1 pulmonary artery pseudoaneurysm. The pulmonary artery pseudoaneurysm was identified intraprocedurally when a 1-cm outpouching was noted along the main pulmonary artery immediately distal to the pulmonic valve. Immediate CTA of the chest confirmed the diagnosis, and no corrective procedure was performed. At 1-month follow-up the pseudoaneurysm was measured and documented stable, and at 1-year follow-up, the pseudoaneurysm was not appreciable on CTA. All four major bleeding or pulmonary vascular injury events observed in the study occurred in patients with intermediate-risk PE.

**Table 4:** Safety outcomes and mortality by risk classification

Outcome	Full cohort *N* = 286	High-risk *N* = 23	Intermediate-high-risk *N* = 215	Intermediate-low- and low-risk *N* = 48
Major bleeding	2 (0.7)	0 (0.0)	2 (0.9)	0 (0.0)
Pulmonary vascular injury	2 (0.7)	0 (0.0)	1 (0.5)	1 (2.1)
Cardiac injury	0 (0.0)	0 (0.0)	0 (0.0)	0 (0.0)
Procedure-associated decompensation	4 (1.4)	3 (13.0)	0 (0.0)	1 (2.1)
New event during or after procedure	0 (0.0)	0 (0.0)	0 (0.0)	0 (0.0)
Exacerbation of ongoing event	4 (1.4)	3 (13.0)	0 (0.0)	1 (2.1)
Intraprocedural cardiac arrest^a^	3 (1.0)	2 (8.7)	0 (0.0)	1 (2.1)
Post-procedural stroke^b^	1 (0.3)	1 (4.3)	0 (0.0)	0 (0.0)
Device-related death	0 (0.0)	0 (0.0)	0 (0.0)	0 (0.0)
7-day all-cause mortality	7 (2.4)	5 (21.7)	2 (0.9)	0 (0.0)
30-day all-cause mortality^c^	19 (6.7)	6 (26.1)	10 (4.7)	3 (6.3)
PE-related death^d^	10 (3.5)	6 (26.1)	3 (1.4)	1 (2.1)
Non-PE-related death	9 (3.1)	0 (0.0)	7 (3.3)	2 (4.2)

Values are *n* (%)

*PE* pulmonary embolism

^a^Both high-risk patients with intraprocedural cardiac arrest also had cardiac arrest prior to procedure. The intermediate-low-risk patient with intraprocedural cardiac arrest had multiple seizures prior to procedure, with an intraprocedural seizure requiring resuscitation

^b^Patient arrested, had impaired neurological exam, and CT suggestive of anoxic brain injury prior to the procedure and during further post-procedural assessments was found to have suffered a stroke. It is possible that the pre-existing neurologic insult was exacerbated by the procedure, though the relatedness is uncertain

^c^*N* = 282 for full cohort and *N* = 211 for intermediate-high-risk PE

^d^All PE-related deaths in the high-risk group occurred in patients who had cardiac arrest prior to the procedure

There were 4 (1.4%) procedure-associated decompensation events, consisting of 3 (1.0%) intraprocedural cardiac arrests and 1 (0.4%) stroke. These 4 events appeared to be exacerbations of ongoing decompensations and not new events that began during or after the procedure. Of the 3 intraprocedural cardiac arrests, 2 occurred in high-risk patients who also experienced cardiac arrest prior to the procedure (1 expired during the procedure and 1 expired in the ICU following the procedure). The remaining intraprocedural cardiac arrest occurred in an intermediate-low-risk patient who had multiple seizures prior to the procedure and an intraprocedural seizure requiring resuscitation; this patient survived to discharge. The stroke occurred in a high-risk patient with arrest, an impaired neurological exam, and CT suggestive of anoxic brain injury prior to the procedure. PE thrombectomy was performed expediently and thereafter, assessment revealed the patient had suffered a stroke. The stroke was considered procedure-associated because the occurrence of intraprocedural paradoxical embolism via a patent foramen ovale could not be ruled out. Twenty patients had clinical decompensation events not associated with the procedure: 5 additional high-risk patients experienced cardiac arrest prior to the procedure, 11 intermediate-risk patients experienced pre-procedural decompensation related to other conditions (cancer *n* = 4, pneumonia *n* = 4, congestive heart failure *n* = 1, sepsis *n* = 1, severe lactic acidosis *n* = 1), and 4 intermediate-risk patients decompensated following the procedure for unrelated reasons (cardiac arrest in the setting of palliative care, hemorrhagic shock, stroke, and adrenal insufficiency; *n* = 1 each). Pre-procedural decompensation in the 4 patients presenting with known cancer diagnosis was attributable to superimposed pneumonia, sepsis, metabolic acidosis, and hemorrhage.

At 7 days post-procedure, all-cause mortality for the full cohort was 2.4% (*n* = 7), including 21.7% (*n* = 5) for patients with high-risk PE, 0.9% (*n* = 2) with intermediate-high-risk PE, and 0% with intermediate-low- or low-risk PE. No deaths were attributed to the device, relationship to the procedure could not be ruled out. All 5 high-risk patients who expired within 7 days had experienced cardiac arrest prior to the procedure and expired while hospitalized. One additional high-risk patient died within 30 days after hospitalization while in hospice; this patient also experienced cardiac arrest prior to the procedure. At 30 days post-procedure, all-cause mortality for the full cohort was 6.7% (*n* = 19), including 26.1% (*n* = 6) for patients with high-risk PE, 4.7% (*n* = 10) with intermediate-high-risk PE, and 6.3% (*n* = 3) with intermediate-low- or low-risk PE. PE-related mortality at 30 days post-procedure was 3.5% (*n* = 10). These PE-related deaths through 30 days included all 6 deaths in high-risk patients, 3 deaths in intermediate-high-risk patients, and 1 death in an intermediate-low-risk patient.

## Discussion

This single-center analysis of LBAT treatment of PE in a large real-world population found that treatment with the FlowTriever System was associated with low 7-day (2.4%) and 30-day (6.7%) all-cause mortality and a favorable safety profile with no cardiac injuries, few pulmonary vascular injuries (0.7%), and minimal procedure-associated decompensations (1.4%). Rapid improvements in hemodynamic measurements were observed.

Patients in this analysis were largely similar in characteristics to those in the all-comer FLASH registry (*N* = 799) [8]. They presented with similar ages (60.5 years in our analysis vs 61.2 years in FLASH), proportion with a history of cancer (18.9% vs 20.7%), proportion with concomitant deep vein thrombosis (62.9% vs 65%), and PE risk stratification (8.0% vs 7.9% high-risk and 90.9% vs 92.1% intermediate-risk). Our analysis also included 3 patients deemed low-risk by the PERT on presentation who were referred to LBAT after failing medical therapy. The concordance between outcomes in this analysis and FLASH suggests that the observation of infrequent major bleeding (0.7% vs 1.4%), common ICU avoidance (49.3% vs 62.6%), and reductions in mean PAP (–6.8 mmHg vs –7.6 mmHg), systolic PAP (–11.7 mmHg vs –12.8 mmHg), and heart rate (–13.5 bpm vs –12.0 bpm) are broadly generalizable. Median total procedure time (83 min vs. 66 min) and post-procedural hospital LOS (5 days vs. 3 days) were increased compared with the FLASH registry [8], but comparable to previously published retrospective analyses [11, 14, 17]. Varying operator experience profiles and study inclusion criteria may have contributed to these differences.

A marginally higher rate of short-term mortality was observed in this analysis than in the FLASH registry (2.4% at 7 days vs 0.3% at 48 h in FLASH) [8]. However, this 7-day all-cause mortality is comparable to the in-hospital mortality found in a recent meta-analysis of aspiration mechanical thrombectomy (3.6%) [18] and may be driven by the inclusion of all LBAT patients, as compared with registry enrollment with certain eligibility criteria. Another recently published, independently conducted retrospective single-center analysis of similar size (*n* = 257) evaluating LBAT with the FlowTriever System reported similar outcomes to those in this study [17]. The overall 30-day mortality rate in the aforementioned study was 3% compared to 6.7% in our analysis, with greater similarity observed in the rate of 30-day PE-attributable mortality (2% compared to 3.5% in this study).

In our analysis, the 7-day mortality rate in high-risk patients was 21.7%. This compares favorably to acute high-risk PE mortality rates in a recent administrative database analysis (39%) and a meta-analysis of high-risk patients (28%) [3, 4]. However, the 7-day mortality rate in high-risk PE in our study appears higher than acute rates reported from the FLASH and FLAME registries (0%–1.9%) [10, 19]. One reason for these differences may lie in the proportion of patients in our analysis who presented with so-called “catastrophic” high-risk PE, a term recently described in the literature [20, 21] and defined as high-risk PE presentation with cardiac arrest or the need for high-dose vasopressors due to concern for impending cardiac arrest. In our analysis, 34.8% (*n* = 8) of high-risk patients experienced cardiac arrest prior to thrombectomy, which would classify them as having catastrophic high-risk PE. In the FLAME and FLASH registries, comparatively fewer high-risk patients experienced cardiac arrest prior to the procedure (20.8% and 6.3%, respectively) [10, 19]. Kobayashi et al. reported that in-hospital mortality among patients with catastrophic high-risk PE was appreciably higher than in non-catastrophic high-risk PE (42.1% vs 17.2%, *p* < 0.001) [21], so the greater prevalence of catastrophic high-risk PE in our analysis may contribute to the higher mortality rates with regard to the FLASH and FLAME registries.

Given our experience with a broad range of intermediate-risk patients, we have found LBAT to be a safe and effective procedure regardless of patient age or anatomy. Technical success can be more difficult to achieve in embolic cases involving chronic thrombus. The key to success is meticulous technique, particularly with guidewire selection and positioning which greatly improve the ability to advance the aspiration catheter to the target location. These same considerations are important to minimize catastrophic guidewire perforations. As our experience has grown, we have been able to carry this same approach to more critically ill, high-risk PE patients. Despite presenting in extremis with cardiac arrest, 25% (*n* = 2) of the “catastrophic” high-risk PE patients treated with LBAT survived through 30-day follow-up. We feel this justifies consideration of the procedure for this subgroup of patients as part of a collaborative PERT discussion.

This study is limited by its retrospective and single-center nature, as well as a lack of long-term outcomes. However, the findings from this large, non-industry-sponsored analysis with no exclusion criteria comprising both high- and intermediate-risk PE patients provide an informative comparison to other FlowTriever clinical studies performed to date.

## Conclusion

This analysis confirms prior clinical study findings that the FlowTriever System is safe and effective for the treatment of high- and intermediate-risk PE in a diverse cohort of PE patients with various comorbidities encountered in the course of real-world clinical practice.
